# Diagnosis of COVID-19 in a Dengue-Endemic Area

**DOI:** 10.4269/ajtmh.20-0676

**Published:** 2020-08-05

**Authors:** Dewi Lokida, Nurhayati Lukman, Gustiani Salim, Deni Pepy Butar-butar, Herman Kosasih, Wahyu Nawang Wulan, Adhella Menur Naysilla, Yuanita Djajady, Rizki Amalia Sari, Dona Arlinda, Chuen-Yen Lau, Muhammad Karyana

**Affiliations:** 1 Tangerang District Hospital, Tangerang, Indonesia;; 2 Indonesia Research Partnership on Infectious Disease (INA-RESPOND), Jakarta, Indonesia;; 3 National Institute of Health Research and Development (NIHRD), Ministry of Health, Jakarta, Indonesia;; 4 National Institute of Allergy and Infectious Diseases (NIAID), National Institutes of Health, Bethesda, Maryland

## Abstract

Emergence of SARS-CoV-2 in dengue virus (DENV)–endemic areas complicates the diagnosis of both infections. COVID-19 cases may be misdiagnosed as dengue, particularly when relying on DENV IgM, which can remain positive months after infection. To estimate the extent of this problem, we evaluated sera from 42 confirmed COVID-19 patients for evidence of DENV infection. No cases of SARS-CoV-2 and DENV coinfection were identified. However, recent DENV infection, indicated by the presence of DENV IgM and/or high level of IgG antibodies, was found in seven patients. Dengue virus IgM and/or high IgG titer should not exclude COVID-19. SARS-CoV-2 reverse transcription polymerase chain reaction (RT-PCR) testing is appropriate when dengue nonstructural protein 1 (NS1) or RT-PCR is negative. Given the possibility of coinfection, testing for both DENV and SARS-CoV-2 is merited in the setting of the current pandemic.

Emergence of SARS-CoV-2 in dengue virus (DENV)–endemic areas has raised concern regarding coinfection with the two viruses.^1,2^ Difficulty in distinguishing dengue and COVID-19, particularly during the acute stage, can engender inaccurate diagnoses. During the COVID-19 pandemic, patients who screen positive on the SARS-CoV-2 questionnaire (Supplemental Table 1) are tested for SARS-CoV-2 by RT-PCR. When confirmed, no further investigation for other etiologies is commonly performed. When SARS-CoV-2 is negative and clinical indication is present (at least fever and thrombocytopenia), DENV NS1 antigen and/or IgM/IgG antibody testing may be performed. Clinicians from Singapore reported two COVID-19 cases that were misdiagnosed as dengue among patients who presented with clinical manifestations and hematology profiles, suggesting dengue infection and false-positive DENV IgM antibody using a rapid diagnostic test (RDT).^3^ This may have occurred because of persistence of DENV IgM from a prior DENV infection. Indonesia has experienced a surge in COVID-19 cases against the backdrop of dengue endemicity. Because the prevalence of DENV IgG antibodies in Singapore is significantly lower than that in Indonesia,^4,5^ we expect Indonesia to face greater challenges with diagnosing SARS-CoV-2, typically performed by RT-PCR, while DENV is co-circulating. To estimate the extent of this problem, we evaluated sera from confirmed COVID-19 patients for evidence of DENV infection.

COVID-19 cases were defined as inpatients who met the COVID-19 criteria based on a predetermined combination of symptoms, laboratory testing, imaging, and risk exposure at Tangerang District Hospital, Indonesia (see Supplemental Table 1), and had a positive nasopharyngeal or oropharyngeal real-time RT-PCR for SARS-CoV-2. Blood and sera were collected from all suspected COVID-19 patients for clinical and research testing. For this study, admission sera for all cases were evaluated for DENV NS1 using RDT (PanBio^®^ Dengue Early Rapid, Abbot, Brisbane, Australia) and ELISA (Dengue NS1 Antigen DxSelect™, Focus Diagnostics, Cypress, CA) assays, and for DENV IgM and IgG using RDT (PanBio^®^ Dengue Duo Cassette, Abbot, Sinnamon Park, Australia) and ELISA (Focus Diagnostics) assays. If available, follow-up sera from 8.5 ± 2.1 days later were evaluated for DENV IgM and IgG by the same RDT and ELISA methods. Admission sera from cases with positive DENV IgM were evaluated using RT-PCR. Results were not returned in real time for patient care purposes.

Clinical and laboratory information on admission was obtained by chart review. Descriptive statistics were performed to characterize the presentation of COVID-19 among these cases and to assess DENV infection status. This research was approved by the Tangerang District Hospital ethics committee.

Admission sera were available for 42 COVID-19 cases. Follow-up sera were available for 32 of these patients. The mean age was 43.5 (SD ± 14.1) years, with a male predominance (66.7%). Time from the onset of illness to serum collection was 8.1 days (SD ± 4.2) days. The most common signs and symptoms were fever (90.5%); cough (83.3%); fatigue, dyspnea, and dysgeusia (38.1% each); sore throat (33.3%); headache (19%); and anosmia and diarrhea (11.9% each). Lymphopenia (< 1,000/mm^3^), leukopenia (< 4,000/mm^3^), and thrombocytopenia (< 150,000/mm^3^) during admission were found in 26.2%, 11.9%, and 4.8%, respectively. A comparison of signs and symptoms for our patients with COVID-19 and dengue patients from a recent fever study^5^ is shown in Supplemental Table 2. None of the 42 subjects was positive for dengue NS1 or showed seroconversion or increasing DENV IgM and IgG index values, suggesting no acute DENV infection among these COVID-19 cases. However, both DENV IgM and high IgG titer, as indicated by positive IgG RDT, were identified in three patients. Dengue virus RT-PCR was negative in all cases. The detection cutoff of IgG PanBio RDT is set at a high titer (equivalent to a hemagglutination inhibition titer ≥ 1,280), which is commonly found in recent secondary DENV infection.^6^ Thus, these cases were probably acute COVID-19 cases with recent secondary DENV infection.

Retrospective assessment of exposure history revealed that one patient could have been infected by SARS-CoV-2 during hospitalization for dengue a week prior at a different hospital. Another patient reported that a clinician he visited before hospitalization suspected DENV infection clinically, but the NS1 result was negative. The third patient did not recall having a fever before acute COVID-19 illness, suggesting asymptomatic or mild dengue, the most common presentation of DENV infection.^7^

In four patients, DENV IgM was detected but did not increase in follow-up samples, DENV IgG was only detected by ELISA, and DENV RT-PCR was negative, suggesting that DENV infection occurred less recently than in the three patients described earlier. Detection of IgM is plausible as it may be detected until 1 year postinfection.^8^ Most patients (33, 78.6%) only had DENV IgG antibodies, implying past DENV infection. This is consistent with previous studies conducted in Indonesia, which demonstrated that more than 90% of adults aged > 18 years had been infected by DENV.^5^ No evidence of DENV infection was identified in two (4.8%) patients. The distribution of dengue diagnostic results is shown in Table 1.

**Table 1:** Interpretation of dengue diagnostic test results among patients with COVID-19

Patients (*N* = 42)	RT-PCR/NS1 antigen	IgM RDT or ELISA	IgG RDT	IgG ELISA	Interpretation
2	NS1 negative	Negative	Negative	Negative	Never infected by DENV
33*	NS1 negative	Negative	Negative	Positive	Past infection by DENV
4	RT-PCR and NS1 negative	Positive (no follow-up IV increase)	Negative	Positive	Recent secondary DENV infection
		#1: 4.8 to 4.1		#1: 9.5 to 9.2	
		#2: 2.8 to 0.5		#2: 8.5 to 8.1	
		#3: 1.6 to 0.8		#3: 4.8 to 6.4	
		#4: 3.7 to 1.6		#4: 10.2 to 9.5	
3	RT-PCR and NS1 negative	Positive (no follow-up IV increase)	Positive faint	Positive	Very recent secondary DENV infection, with high-titer IgG
		#1: 3.8 to 3.8		#1: 17.1 to 16.6	
		#2: 2.5 to 1.1		#2: 12.3 to 11.5	
		#3: 1.6 to 1.5		#3: 12.5 to 12.3	

DENV = dengue virus; IV = index value; RDT = rapid diagnostic test.

* Ten patients in this group did not have follow-up sera. For patients with positive IgM and IgG, admission and follow-up IV are shown. Changes in both IgM and IgG interpretation were not noted in follow-up sera. There were no cases of current dengue coinfection.

Despite concurrent high incidence of COVID-19 and dengue in Indonesia, acute coinfection with DENV was not detected in this cohort of patients identified to have COVID-19. This may be because of SARS-CoV-2 and DENV testing practices, which focus on symptomatic cases. It is likely that coinfection is occurring but is often asymptomatic. It is also possible that some patients had already been infected with current circulating DENV serotypes and thus had immunity, as indicated by high prevalence of patients with IgG antibodies.

Identification of seven (16.7%) COVID-19 cases with DENV IgM in our cohort may be related to the occurrence of COVID-19 during the yearly dengue season. This finding is concerning, particularly because clinicians frequently diagnose dengue based only on DENV IgM, which may persist for months after resolution of infection. Our study demonstrates that adding NS1 to the diagnostic algorithm may reduce dengue overdiagnosis attributable to reliance on IgM. Missed diagnosis of acute COVID-19 due to presumption of dengue can result in inadvertent omission of targeted precautions, which could lead to transmission to contacts, including family, colocated patients, and healthcare workers. A missed diagnosis could also delay the receipt of standard of care COVID-19 treatment. Missed diagnoses have been reported in Singapore due to false-positive DENV IgM RDT results^3^ versus persistence of DENV IgM. In the setting of the current pandemic and in light of overlapping symptomatology, clinicians should test for both DENV and SARS-CoV-2.

It is notable that one of the patients may have contracted SARS-CoV-2 during hospitalization a week prior. In resource-limited settings, adequate infection control practices are difficult to implement. Hence, nosocomial infection should be considered in the setting of recent contact with the healthcare system, including due to dengue. SARS-CoV-2 infection control strategies for resource-limited settings are needed.

Findings from our study should be interpreted with caution. The study population was small and from only one hospital at Tangerang district, during March 2020 and April 2020. Therefore, results have limited generalizability as they reflect the epidemiology of COVID-19 and dengue in that area during the study period. Furthermore, as the assays were qualitative (RDT) or semi-quantitative (ELISA), increasing antibody titer was only measured by the index value, which may be inaccurate. To reduce inaccuracy, we tested acute and follow-up specimens simultaneously.

In conclusion, our study reaffirms challenges associated with diagnosing COVID-19 in areas hyperendemic for tropical infections with overlapping presentations such as dengue. The known potential for repeat dengue infections and the possibility for repeat SARS-CoV-2 infections add further complication. When molecular diagnostic testing for DENV is not available, we recommend the use of a validated NS1 and IgM/IgG RDT. Addition of NS1 will improve the specificity of identifying acute dengue cases.^9^ Detection of DENV IgM and/or high IgG titer should not be considered an exclusion of COVID-19. Past infection with DENV with acute COVID-19 or even acute DENV and SARS-CoV-2 coinfection would remain possibilities.^10^ Hence, evaluation for COVID-19 should be conducted when dengue NS1 or RT-PCR (when available) is negative.

## Supplemental tables

Supplemental materials
