# Supplementary material for: Diagnosis of COVID-19 in a Dengue-Endemic Area
Source: Am J Trop Med Hyg. 2020 Aug 5;103(3):1220–2. doi: 10.4269/ajtmh.20-0676 (PMC7470577; doi:10.4269/ajtmh.20-0676)
Supplement: Supplementary file 1 [file tpmd200676.SD1.pdf]

Supplemental Table 1. COVID-19 Screening Form Tangerang District Hospital

Patient's Name : ……………………………………………………………………………………..
Date of birth / Age : ……………………………………………………………………………………..
Address : ……………………………………………………………………………………..
Phone : ……………………………………………………………………………………..

No	Questions	Yes	No
**Symptoms**			
1	Fever / fever history		
2	Signs and symptoms of respiratory disorders: cough, cold/flu, sore throat, etc.		
3	Pneumonia / severe acute respiratory infections (hard to breathe)		
4	Diarrhea / myalgia		
5	No other causes based on convincing clinical descriptions		
**Supporting Examinations**			
1	Hemoglobin ≥ 10 g%		
2	Leukopenia		
3	Lymphopenia		
4	CXR: bilateral and peripheral opacity		
5	CT Scan: ground glass opacities		
**Underlying Conditions**			
1	Age >65 years		
2	Lung disease: TBC, asthma, Chronic Obstructive Pulmonary Disease (COPD)		
3	Cardiovascular disease: hypertension, heart disease		
4	Diabetes Mellitus (DM)		
5	Cancer / ………………………..		
**Risk Factors**			
1	Contact history with confirmed covid-19 cases in the last 14 days?		
2	Been/travelled abroad where local transmission was occuring in the last 14 days?<br>o China<br>o Italia<br>o Iran<br>o Spain<br>o South Korea<br>o Germany<br>o France<br>o Other. Specify: ………………………..		
3	Travelled domestically where local transmission was occurring in the last 14 days?		

	o Jakarta<br>o Depok<br>o Bekasi<br>o Tangerang<br>o Solo<br>o Other. Specify: ………………………..		
**Assessment Method**			
**Patient Under Observation / *Pasien Dalam Pengawasan (PDP)***	- Symptom 1 + 2 + 3 + 4 + 5 and Risk Factor (RF) 1 or 2 or 3<br>- Symptom 1 or 2 or 3 and RF 1<br>- Symptom 1 and/or 2 + Supporting examination 2 + 3<br>- Symptom 1 or 2 or 3 + Supporting examination 4 or 5		
**People Under Observation / *Orang Dalam Pengawasan (ODP)***	Symptom 1 or 2 or 3 and RF 2 or 3		
**Close Contact High Risk**	RF 1		
**Follow-Up Plan**			

Put tick (√) in the appropriate box!

Date : ________________________

Doctor's Signature
