# Supplementary material for: Diagnosis of COVID-19 in a Dengue-Endemic Area
Source: Am J Trop Med Hyg. 2020 Aug 5;103(3):1220–2. doi: 10.4269/ajtmh.20-0676 (PMC7470577; doi:10.4269/ajtmh.20-0676)
Supplement: Supplementary file 2 [file tpmd200676.SD2.pdf]

Supplemental Table 2. Comparison of signs and symptoms of patients with COVID-19 and dengue

Clinical signs and symptoms	COVID-19 (n=42)	Dengue (n=468)
Fever	37 (88)	468 (100)
Headache	13 (31)	261 (56)
Vomiting	8 (19)	249 (53)
Cough	34 (81)	79 (17)
Diarrhea	7 (17)	47 (10)
**Hematology findings: Median (range)**		
Leucocyte count: /mm3	5,950 (1,400-11,300)	3,600 (800-20,100)
Leukopenia	5 (12)	312 (67)
Leukocytosis	3 (7)	9 (2)
Lymphocyte (%)	25 (7-57)	25.5 (4-77,8)
Lymphocyte absolute	1,317 (476-3,185)	888 (192-7,104)
Lymphocytopenia	14 (33)	110/414 (27)
Lymphocytosis	3 (7)	84/414 (20)
Platelet count	247,500 (100,000-600,000)	91,900 (10,000-452,000)
Platelet < 100,000/ mm3	0 (0)	266 (57)
Platelet < 150,000/ mm3	2 (5)	373 (80)

Thrombocytopenia (150,000) AND leukopenia (<5000)	1 (2)	318 (68)
